# The prospects of working memory training for improving deductive reasoning

**DOI:** 10.3389/fnhum.2015.00056

**Published:** 2015-02-06

**Authors:** Erin L. Beatty, Oshin Vartanian

**Affiliations:** ^1^Defence Research and Development Canada, Toronto Research CentreToronto, Canada; ^2^Department of Psychology, University of Toronto ScarboroughToronto, Canada

**Keywords:** reasoning, working memory training, fluid intelligence, n-back, education intervention

Cognitive (brain) training has been a major focus of study in recent years. In applied settings, the excitement regarding this research programme emanates from its prospects for *far transfer*—defined as observing performance benefits in outcome measures that are contextually, structurally or superficially dissimilar to the trained task (Perkins and Salomon, 1994). By and large, researchers have focused on training working memory (WM). This is not surprising, given the ubiquity of WM requirements for thinking (Baddeley, 2003). Currently, much evidence suggests that adaptive training on WM tasks can increase WM skills. In contrast, consistent evidence regarding far transfer is lacking (see Melby-Lervåg and Hulme, 2013), although there is evidence to suggest that when the training modality is visuospatial, the likelihood of transfer and the long-term stability of its benefits are enhanced (Melby-Lervåg and Hulme, 2013; Stephenson and Halpern, 2013).

Theoretically, there is reason to suspect that interventions that increase WM skills and/or capacity could improve deductive reasoning. This prediction stems from the observation that individual differences in WM capacity predict deductive reasoning performance on conflict problems where the believability of conclusions conflicts with logical validity (e.g., Newstead et al., 2004). Conflict problems require WM resources because their correct solution depends on the suppression of the heuristic system (System I) in favor of responding in accordance with the analytic system (System II). Evidence for this interpretation was provided by De Neys (2006), who presented participants with conflict and non-conflict syllogisms while also burdening their executive resources with a secondary task. Specifically, the between-subjects manipulation of WM load consisted of presenting a 3 × 3 matrix prior to each syllogism, wherein the matrix was filled with a complex four-dot pattern (high load) or with three dots on a horizontal line (low load)^1^. After making a validity judgment, participants reproduced the matrix pattern. This experimental design required them to maintain the matrix pattern in WM while reasoning. Whereas the high load condition impaired performance on conflict problems, there was no effect of load on non-conflict problems. This demonstrates that overcoming belief-logic conflict is limited by WM capacity.

WM training could also lead to improvement in deductive reasoning via its effect on fluid intelligence—typically measured using matrix reasoning tasks. Specifically, much evidence suggests that general cognitive ability and deductive reasoning are positively correlated (Stanovich and West, 2000). In addition, a recent meta-analysis demonstrated that training specifically on the *n*-back family of WM tasks leads to a small but positive effect on fluid intelligence (Au et al., 2014). Therefore, theoretically, increases in fluid intelligence could mediate the link between *n*-back training and deductive reasoning, offering an indirect route for improving the latter (Figure 1).

**Figure 1:**
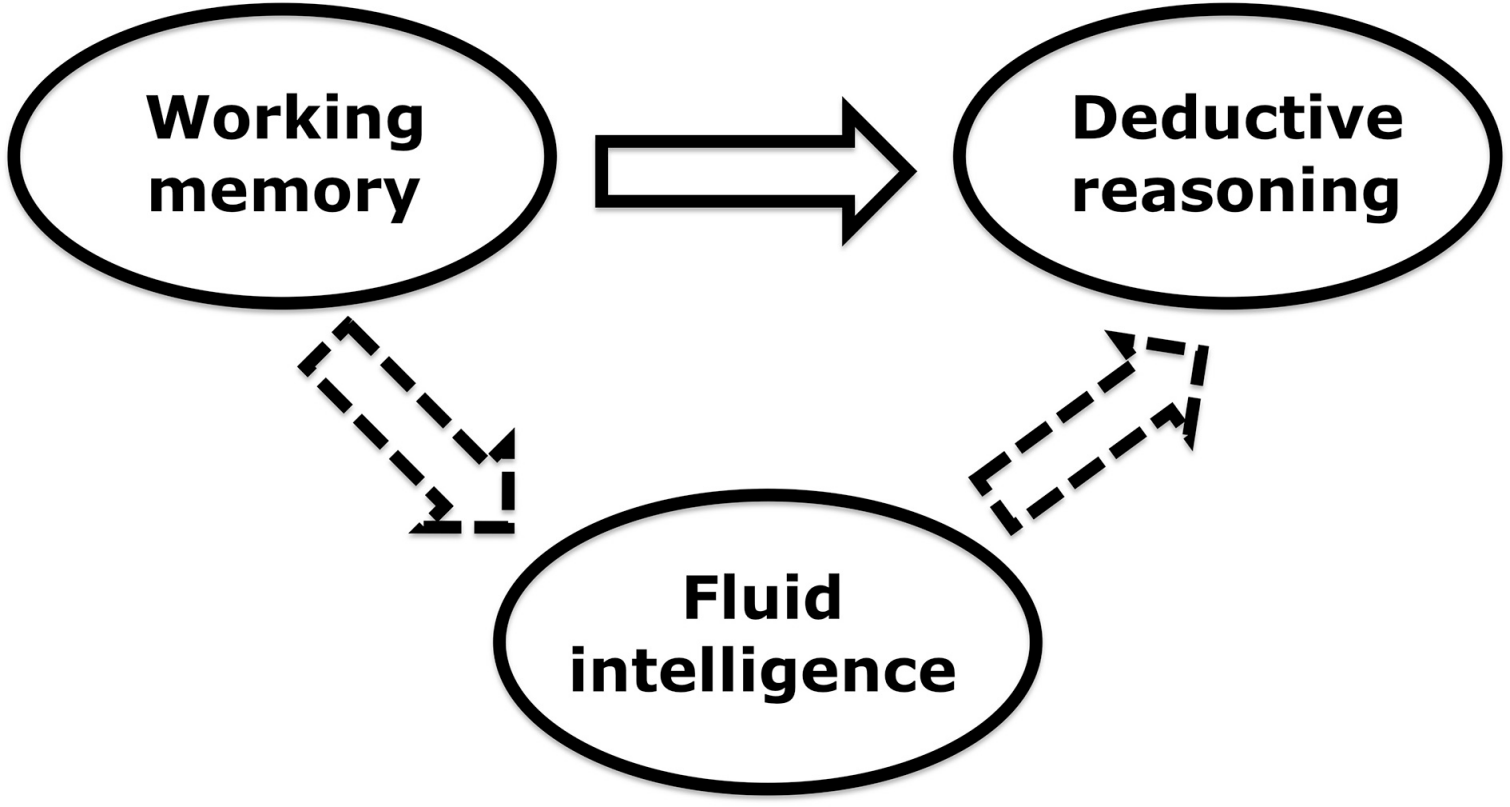
**Two possible routes for improving deductive reasoning by working memory training**. The solid arrow depicts a direct effect. The dashed arrows depict an indirect effect.

Recently, Ariës et al. (2014) investigated the combined effect of reasoning strategy and WM training on school performance. The participants for Experiment 1 were enrolled in *lower-level* Higher Secondary Education history classes. During the 6-week intervention period, participants in the control condition were taught using a “conservative” method that involved the introduction of new subjects in new paragraphs, and the answering of reasoning questions from the textbook. In contrast, for participants in the experimental condition the same material was embedded within two WM training tasks: *n*-back and the Odd One Out. This approach ensured that training was contextualized within the subject matter of the history class. For example, on each trial of the Odd One Out four historical words or pictures were presented successively on the screen, three of which were related (e.g., were drawn from agrarian civilizations) whereas the fourth was not (i.e., was a depiction of hunter-gatherer civilization). The participant had to maintain all four stimuli in WM to select the odd one out. In the *n*-back task, nouns (e.g., farming) and pictures (e.g., hieroglyphics) drawn from the content of the history class were used as stimuli.

In addition, the experimenters trained reasoning strategies using a modification of the IMPROVE method (see Mevarech and Kramarski, 2003). This intervention is designed to teach the structure of reasoning, and works by testing understanding of the problems, highlighting similarities between problems, applying strategies for solving problems, and prompting reflection on the reasoning process. Compared to the control condition, students in the experimental condition exhibited significant gains in performance on reasoning questions in official school tests that necessitate inference making—a difference that remained significant 16 weeks after the termination of training. Subsequently, participants in Experiment 2 who were enrolled in *higher-level* Higher Secondary Education history classes received *either* WM *or* reasoning strategy training. On its own, reasoning strategy but not WM training improved school test performance.

The results of Ariës et al. (2014) suggest that for students of relatively lower ability, the combination of WM and reasoning strategy training can be a successful recipe for improving reasoning. This is likely because whereas the former enhances WM skills, the latter facilitates the acquisition of the cognitive tools for logic. For students of higher ability there might be less room for improving WM (i.e., a ceiling effect), such that learning the structure of reasoning becomes a relatively more important factor for improving performance. Although the results of the two experiments are not directly comparable because of differences in the composition of the samples and intervention strategies, they do suggest that differences in baseline ability must be taken into account while assessing transfer effects (see Jaeggi et al., 2014).

In conclusion, it appears useful to pursue the possibility that WM training could benefit deductive reasoning directly by increasing WM skills, or indirectly by increasing fluid intelligence. Critically, Ariës et al.'s successful intervention consisted of embedding WM training with domain-relevant material. It has yet to be demonstrated whether a domain-general intervention to train WM will exhibit a similar transfer profile in the context of deductive reasoning. In addition, the extent to which successful transfer to deductive reasoning will require supplementing WM training with strategy training remains an open question.

## Conflict of interest statement

The authors declare that the research was conducted in the absence of any commercial or financial relationships that could be construed as a potential conflict of interest.

## Footnotes

^1^There was also a third no-load condition—not pertinent to the present discussion.
